# Dynamic Changes in Microorganisms and Metabolites During Silage Fermentation of Whole Winter Wheat

**DOI:** 10.3390/vetsci12080708

**Published:** 2025-07-28

**Authors:** Li Zhang, Yu Zeng, Lin Fu, Yan Zhou, Juncai Chen, Gaofu Wang, Qifan Ran, Liang Hu, Rui Hu, Jia Zhou, Xianwen Dong

**Affiliations:** 1 Chongqing Academy of Animal Sciences, Chongqing 402460, China; zhangli03094@163.com (L.Z.); zycqxky@163.com (Y.Z.); lyfl1990@163.com (L.F.); zhouyanxky@163.com (Y.Z.); wanggaofs20031216@163.com (G.W.); ranqifan@outlook.com (Q.R.); 2 College of Animal Science and Technology, Southwest University, Chongqing 400715, China; juncaichen@swu.edu.cn; 3 Wushan County Animal Husbandry and Aquatic Technology Service Center, Chongqing 404700, China; huliang5210@163.com; 4 Animal Nutrition Institute, Sichuan Agricultural University, Chengdu 611130, China; hurui14648@sicau.edu.cn

**Keywords:** metabolic dynamics, fermentation quality, silage, microbiome, organic acid

## Abstract

Winter wheat (*Triticum aestivum* L.) silage has high feeding value and has become an important roughage resource in China. To recognize the optimal fermentation time of the silage product, this study systematically evaluated the temporal dynamics of microbial communities and metabolic profiles in whole winter wheat silage at days 7, 14, 30, 50, and 70. The dry matter (DM) content slightly fluctuated with the extension of fermentation time, with 28.14% at 70 days of ensiling. The organic matter and neutral detergent fiber content gradually decreased with the extension of fermentation time. A significant decrease in pH was observed at days 30, 50, and 70 compared to days 7 and 14 (*p* < 0.05), with the lowest pH value of 4.4 recorded at day 70. The contents of lactic acid, acetic acid, butyric acid, and total volatile fatty acids gradually increased with the extension of fermentation time, reaching a maximum at 70 days of ensiling. The dominant bacteria were Proteobacteria and Firmicutes at the phylum level, and the predominant bacteria were *Hafnia-Obesumbacterium*, *Enterobacter*, and *Lactobacillus* at the genus level. The relative abundance of *Hafnia-Obesumbacterium* and *Lactobacillus* fluctuated slightly with the duration of fermentation, reaching a minimum for the former and a maximum for *Lactobacillus* at 50 days of ensiling. By day 70, *Sporolactobacillus* emerged as a distinct silage biomarker. The dominant fungi was Ascomycota at the phylum level, and the predominant fungi were *Fusarium* and an unidentified fungus at the genus level. The correlation analysis revealed significant pH–organic acid–microbe interactions, with pH negatively correlating with organic acids but positively with specific bacteria, while organic acids showed complex microbial associations. Collectively, under natural fermentation conditions, the optimal fermentation period for wheat silage exceeds 70 days, and *Sporolactobacillus* shows potential as a microbial inoculant for whole winter wheat silage. These findings provide a theoretical foundation for optimizing whole winter wheat silage utilization and enhancing fermentation quality.

## Simple Summary

This study investigated how fermenting whole winter wheat as animal feed improves its storage quality and nutritional value over time, monitoring changes in the silage over 70 days. The results showed that fermentation increased beneficial acids while reducing spoilage microbes, enhancing the silage’s stability and nutritional content. These findings offer farmers a practical method to improve animal nutrition using locally grown crops, as fermented whole winter wheat serves as a sustainable feed option that can boost productivity and reduce costs, ultimately benefiting livestock producers and the broader food system through more efficient resource utilization.

## 1. Introduction

With the exponential growth of the global population and the escalating demand for food supplies, the efficient utilization of agricultural resources and the reduction of food waste have emerged as critical issues in contemporary agricultural research. Currently, approximately 40% of arable land in the global food system is dedicated to animal feed production [1,2], a significant proportion that highlights the pivotal role of livestock in worldwide agricultural practices. However, this resource allocation paradigm has ignited extensive academic discourse regarding food security and land utilization efficiency. Particularly in China, despite significant progress in animal husbandry, the proportion of artificial grassland relative to total pasture or arable land remains considerably lower than the global average [3,4]. This distinctive phenomenon underscores not only the uniqueness of China’s agricultural framework but also the significant challenges within its feed production systems and sustainable land management practices.

In China, farmland after the autumn harvest experiences a long fallow period (from October to following April), resulting in insufficient utilization of available arable land resources [5]. Maximizing the use of winter fallow fields for cultivating feed crops, such as winter wheat, is expected to mitigate shortages in China’s livestock industry. The annual winter wheat production in China reaches around 111 million ton, predominantly cultivated in the northern plains [6,7]. As a crucial feed preservation technique, silage technology significantly extends feed storage duration while minimizing nutritional losses [8], thereby enhancing feed utilization efficiency in livestock production. Winter wheat demonstrates superior natural fermentation potential due to its remarkable adaptability to varying environmental conditions, optimal dry matter (DM) content, and high water-soluble carbohydrate (WSC) levels that promote lactic acid bacterial growth and activity [6,9]. This characteristic endows winter wheat with considerable potential for enhancing silage production through optimized fermentation processes. Comparative analysis demonstrated that whole wheat silage exhibited significantly higher concentrations of ethanol-soluble carbohydrates, crude protein (CP), soluble CP, and essential mineral elements including calcium (Ca), phosphorus (P), and potassium compared to corn silage [10]. Studies have revealed significant alterations in the chemical composition of whole wheat silage during fermentation, with notable changes observed specifically in WSC and neutral detergent fiber (NDF) levels [11]. However, the current systematic understanding of microbial community dynamics and their associated metabolic transformation mechanisms during winter wheat silage remains incomplete, which seriously restricts the optimization of silage feed quality and nutritional value.

Microbial communities, particularly lactic acid bacteria, serve as fundamental drivers in the silage fermentation process, orchestrating critical biochemical transformations [12]. However, this essential process is frequently compromised by mycotoxin contamination (secondary metabolites produced by filamentous fungi), which substantially deteriorates both the nutritional value and safety parameters of silage products [13]. Recent advancements in silage science have demonstrated that the strategic application of either exogenous fibrolytic enzymes or specifically formulated microbial inoculants can significantly enhance fermentation efficiency and nutritional preservation [14,15,16]. These findings underscore the necessity of elucidating the complex dynamics of microbial communities and their metabolic interactions during the ensiling process to optimize silage quality. Thus, the present study systematically investigates the temporal dynamics of microbial communities throughout the ensiling process, aiming to establish a scientific foundation for the improvement of winter wheat silage fermentation technologies.

## 2. Materials and Methods

### 2.1. Materials and Silage Treatments

Winter wheat (*Triticum aestivum* L. Yu 1768) was cultivated in Wushan County, Chongqing, China (109.88° E, 31.07° N), with sowing conducted on 19 October 2020 and harvesting at the grain filling stage (5.13 ± 0.64 kg/m^2^ fresh weight, 100.03 ± 1.23 height) on 25 April 2021. The comparative analysis of soil composition parameters before and after winter wheat cultivation is presented in Table 1. Fresh forage samples (800 g) with a moisture content of 71.31% were uniformly packed into food-grade polyethylene bags (25 cm × 35 cm, China) following standardized procedures. Subsequently, the bags were vacuum-sealed using a commercial vacuum packaging machine (Model DZ-400, Hualin Packaging Machinery Co., Ltd., Shanghai, China) to maximize air removal prior to final sealing. A total of 80 sealed bags were preserved at room temperature (25–30 °C). The temporal changes in chemical composition, fermentation characteristics, and microbial community (bacterial and fungal) were analyzed in six randomly selected silage samples at five designated time points (7, 15, 30, 50, and 70 days) during the ensiling process.

### 2.2. Chemical Composition Measurement

Both raw materials and ensiled samples underwent drying in a forced-air oven at 65 °C for 72 h, followed by weighing and grinding through a 1 mm sieve for subsequent chemical analyses. Dry matter and crude ash contents were determined following the official protocols established by the Association of Official Analytical Chemists [17]. Organic matter (OM) content was calculated by subtracting crude ash from DM. Total nitrogen (N) content was quantified using a Kjeldahl nitrogen analyzer (KjelFlex K-360, Büchi Labortechnik AG, Flawil, Switzerland) according to the AOAC method, with CP content calculated using the standard conversion factor (total N × 6.25). Ether extract (EE) content was determined through Soxhlet extraction [17]. Neutral detergent fiber and acid detergent fiber (ADF) contents were analyzed according to Van Soest’s methodology [18] using an automated fiber analyzer (FibertecTM 2010, Foss Analytical A/S, Hillerød, Denmark).

### 2.3. Fermentation Quality Measurement

Accurately weighed 20 g of ensiled samples were homogenized with 180 mL of double-distilled water using a laboratory blender for 2 min, followed by filtration through a four-layer medical gauze [19]. The resulting filtrate was immediately subjected to pH measurement using a calibrated pH meter (PHSJ-5; LEICI, Shanghai, China) and organic acid analysis. Lactic acid content was quantified according to the spectrophotometric method as previously described [20]. The concentrations of volatile fatty acids (VFAs), including acetic acid, propionic acid, and butyric acid, were determined using a Varian CP-3800 Gas Chromatograph (Agilent Ltd., Cork, Ireland).

### 2.4. Bacterial and Fungal DNA Amplification and Amplicon Library Preparation

Total genomic DNA was extracted from ensiled samples using the E.Z.N.A.^®^ DNA Kit (Omega Bio-tek, Norcross, GA, USA) according to the manufacturer’s instructions. DNA concentration and purity were assessed using a NanoDrop 2000 spectrophotometer (Thermo Fisher Scientific, Wilmington, DE, USA) and 1% (*w*/*v*) agarose gels electrophoresis. Qualified DNA samples were aliquoted and stored at −20 °C for subsequent molecular analyses.

The V3-V4 hypervariable regions of bacterial 16S rRNA genes were amplified using specific primers 338F (5′-ACTCCTACGGGAGGCAGCAG-3′) and 806R (5′-GGACTACNNGGGTATCTAAT-3′), while the ITS2 region of fungal ribosomal DNA was amplified using primers ITS3-KYO2 (5′-GATGAAGAACGYAGYRAA-3′) and ITS4 (5′-TCCTCCGCTTATTGATATGC-3′). Unique 8-bp barcode sequences were incorporated at the 5′ end of both forward and reverse primers for each sample to enable sample multiplexing during sequencing, with all primers synthesized by Allwegene Technologies Co., Ltd. (Beijing, China).

PCR amplification was performed in 25 μL reactions using an ABI 9700 thermal cycler (Applied Biosystems, Foster, CA, USA) with the following components: 12.5 μL of 2× Taq PCR MasterMix (Vazyme Biotech Co., Ltd., Nanjing, China), 1 μL of each primer (10 μM), 2 μL of template DNA (10 ng/μL), and 8.5 μL of nuclease-free water. The thermal cycling protocol was programmed as follows: initial denaturation at 95 °C for 5 min; 28 cycles of denaturation at 95 °C for 45 s, annealing at 55 °C for 50 s, and extension at 72 °C for 45 s, followed by a final extension at 72 °C for 10 min. The PCR products were purified using the Agencourt AMPure XP magnetic bead system (Beckman Coulter, Brea, CA, USA) following the manufacturer’s protocol. Sequencing libraries were constructed using the NEB Next Ultra II DNA Library Prep Kit (New England Biolabs, Ipswich, MA, USA) according to the manufacturer’s instructions. Library quality was assessed through a three-step process: (1) quantification using a NanoDrop 2000 spectrophotometer (Thermo Fisher Scientific); (2) size distribution analysis with an Agilent 2100 Bioanalyzer (Agilent Technologies, Santa Clara, CA, USA); and (3) quantification and amplification efficiency validation by qPCR using an ABI StepOnePlus Real-Time PCR System (Applied Biosystems). Finally, the libraries were sequenced on an Illumina NovaSeq 6000 platform (Illumina, Inc., San Diego, CA, USA) using a PE250 strategy following standard protocols.

### 2.5. Data Analysis of Microbial Diversity

The raw sequences were demultiplexed using barcode sequences and processed with PEAR [21] to remove low-quality sequences (quality score ≤ 20) and ambiguous bases (N), with assembly parameters set at 10 bp minimum overlap and *p*-value of 0.0001. Further processing used Vsearch [22]: bacterial sequences < 230 bp were removed, and chimeras were filtered using uchime based on the Gold Database (https://gold.jgi.doe.gov/, accessed on 26 June 2020); fungal sequences <120 bp (ITS2 retained at 230 bp) were removed, and chimeras were filtered using uchime referencing the Unite database (https://unite.ut.ee/, accessed on 27 June 2020) [23]. Qualified sequences were clustered into OTUs at 97% similarity using the Uparse [24] algorithm in Vsearchv [22]. Taxonomic classification was performed using BLAST (v2.15.0) [25] against the Silva138 Database (for bacterial 16S) and Unite8.2 Database (for fungal ITS), with an e-value threshold of 1*10^−5^. Alpha diversity analysis (rarefaction curves, richness, and diversity indices) was conducted in QIIME (v1.8.0) and visualized in R (v3.6.0). Taxonomic composition was displayed using bar plots, and beta diversity was assessed via PCA and Bray–Curtis distance matrices, visualized through PCoA or UPGMA clustering trees in R (v3.6.0). Finally, LEfSe analysis was carried out using Python (v2.70) to identify differentially abundant taxa across sample groups [26].

### 2.6. Statistical Analysis

Statistical analyses were performed using SPSS Statistics 26.0 (IBM Corp., Armonk, NY, USA). Chemical composition, fermentation quality parameters, and alpha diversity indices of microbial communities were analyzed by one-way analysis of variance (ANOVA) followed by Duncan’s multiple comparison test. Statistical significance was set at *p* < 0.05. Graphical representations were generated using GraphPad Prism 9.5 (GraphPad Software, San Diego, CA, USA). Spearman’s correlation analysis between microbial diversity and fermentation quality metrics was conducted using the Omicshare platform (https://www.omicshare.com/tools/, accessed on 15 January 2025).

## 3. Results

### 3.1. Chemical Composition

**Figure 1:**
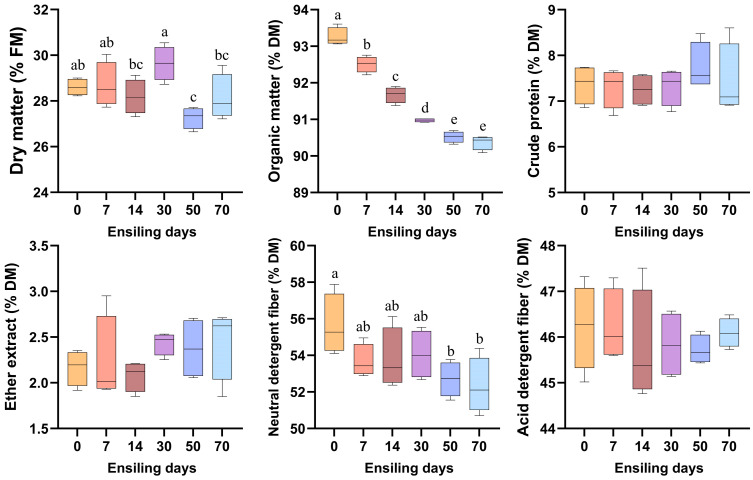
Temporal dynamics of chemical composition in fermented winter wheat during ensiling. FM, fresh matter; DM, dry matter. Different lowercase letters indicate *p* < 0.05, n = 6.

As illustrated in Figure 1, the DM content demonstrated significant temporal variations throughout the ensiling process (*p* < 0.05), peaking on day 30 and reaching its minimum on day 50, which was significantly lower than the initial value at day 0. The OM content progressively decreased during ensiling, with the maximum level observed at day 0 and stabilizing after day 50. Notably, the NDF content on days 50 and 70 showed a significant reduction compared to day 0 (*p* < 0.05). In contrast, the contents of CP, EE, and ADF remained statistically unchanged across all sampling time points (*p* > 0.05).

### 3.2. Fermentation Quality Parameters

**Figure 2:**
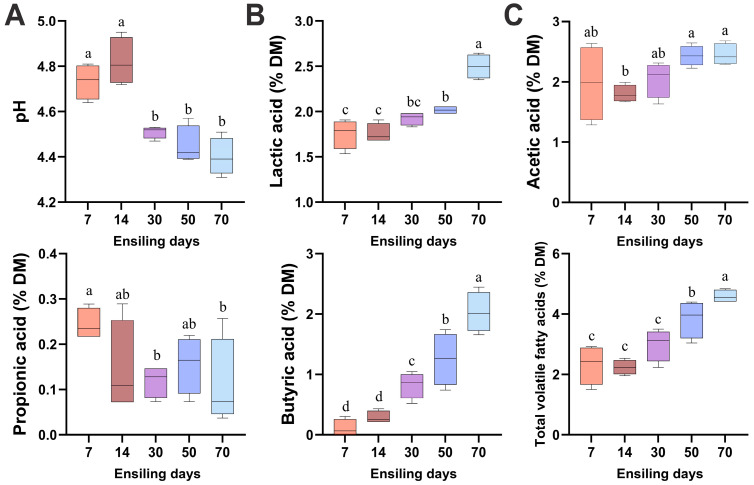
Temporal dynamics of pH (**A**), lactic acid level (**B**), and volatile fatty acids (**C**) in fermented winter wheat during ensiling. DM, dry matter. Different lowercase letters indicate *p* < 0.05, n = 6.

As illustrated in Figure 2, the pH values of winter wheat silage were significantly lower on days 30, 50, and 70 compared to days 7 and 14 (*p* < 0.05). The concentrations of lactic acid, butyric acid, and total VFAs in winter wheat silage progressively increased throughout the fermentation process, reaching their maximum levels on day 70. The acetic acid content showed an increasing trend during the ensiling process, with significantly higher concentrations observed on days 50 and 70 compared to day 14 (*p* < 0.05). Conversely, propionic acid content exhibited a decreasing trend, demonstrating lower concentrations at days 30 and 70 than at day 7 (*p* < 0.05).

### 3.3. Microbial Community Analysis of Silage

**Figure 3:**
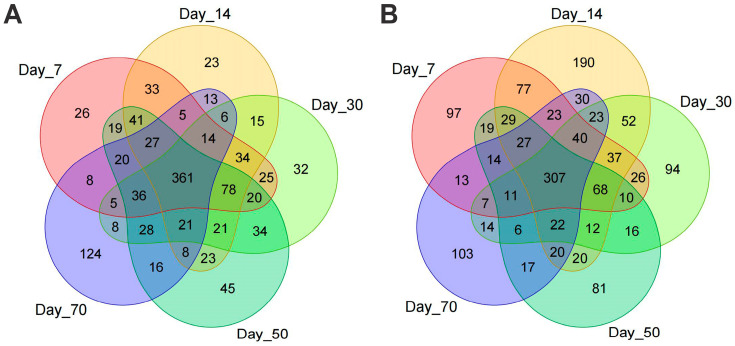
Venn diagrams of OTUs across the five treatments for bacterial (**A**) and fungal (**B**) communities.

A total of 4,095,331 high-quality sequencing reads were obtained, comprising 1,505,224 reads from the 16S rRNA gene (bacterial community) and 2,590,107 reads from the ITS region (fungal community), for subsequent microbial community analysis. These sequences were clustered into operational taxonomic units (OTUs) at a 97% sequence identity threshold, yielding 1169 bacterial OTUs and 1505 fungal OTUs. Among these, 361 bacterial OTUs and 307 fungal OTUs were shared across all five time points, representing 30.88% and 20.40% of the total effective sequences for the 16S rRNA and ITS datasets, respectively (Figure 3).

**Figure 4:**
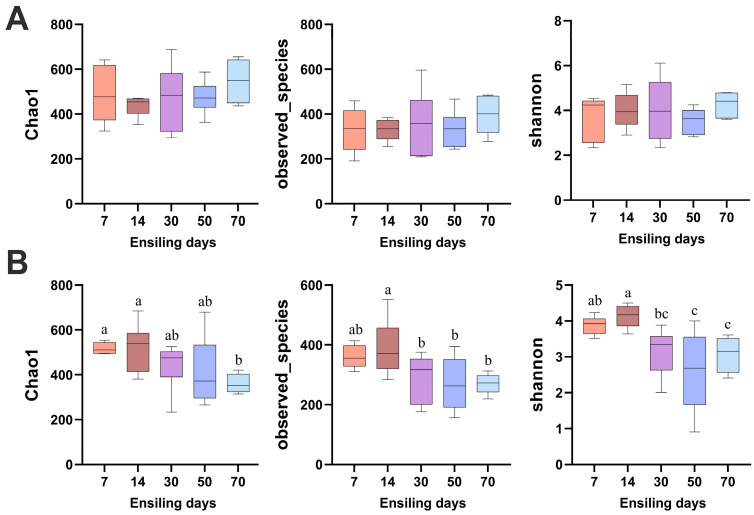
Alpha diversity analysis of bacterial (**A**) and fungal (**B**) communities in winter wheat silage at different fermentation stages (7, 14, 30, 50, and 70 days). Different lowercase letters indicate *p* < 0.05, n = 6.

The richness of bacterial and fungal communities in the silage, as assessed by the observed species and Chao1 indices, as well as the diversity estimated by the Shannon index, are presented in Figure 4. The bacterial alpha diversity showed no significant differences across the various time points (*p* > 0.05). In contrast, the fungal diversity exhibited a significant decreasing trend over the ensiling period. Specifically, at day 70, the fungal Chao1 index was significantly lower than that at days 7 and 14 (*p* < 0.05). The observed species at days 30, 50, and 70 were significantly lower than at day 14 (*p* < 0.05), and the Shannon index at days 50 and 70 was significantly lower than at days 7 and 14 (*p* < 0.05).

**Figure 5:**
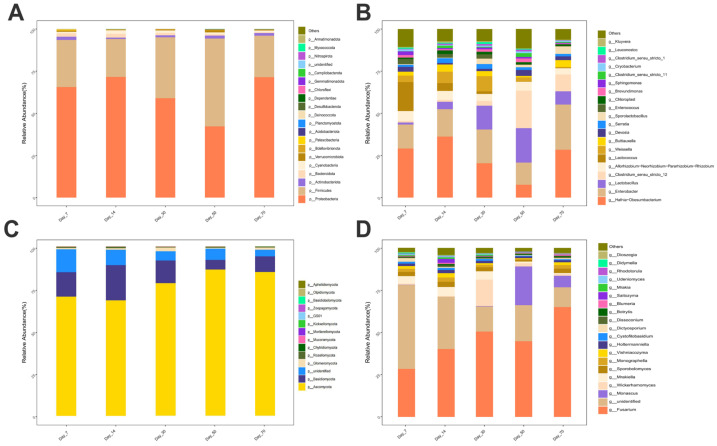
Relative abundance of bacterial and fungal communities in winter wheat silage at the genus (**A**,**C**) and phylum (**B**,**D**) levels across different fermentation stages (7, 14, 30, 50, and 70 days).

A total of 20 bacterial and 14 fungal phyla were identified across all samples, with seven phyla shared between the two communities (Figure 5A,C). At the phylum level, the two most abundant bacterial phyla were Proteobacteria and Firmicutes, representing an average relative abundance of 61.98% and 32.66%, respectively. Similarly, the two most abundant fungal phyla were Ascomycota and Basidiomycota, accounting for 77.71% and 12.73% of the total fungal community, respectively. At the genus level, 22 bacterial and 20 fungal genera were identified (Figure 5B,D). The four most abundant bacterial genera were *Hafnia-Obesumbacterium*, *Enterobacter*, *Lactobacillus*, and *Clostridium_sensu_stricto_12*, contributing 24.35%, 18.09%, 9.59%, and 7.46% to the total bacterial community, respectively. The three most abundant fungal genera identified, in descending order, were *Fusarium*, *Monascus*, and *Wickerhamomyces*, with average relative abundances of 45.74%, 6.02%, and 3.90% of the total fungal community at the genus level, respectively.

**Figure 6:**
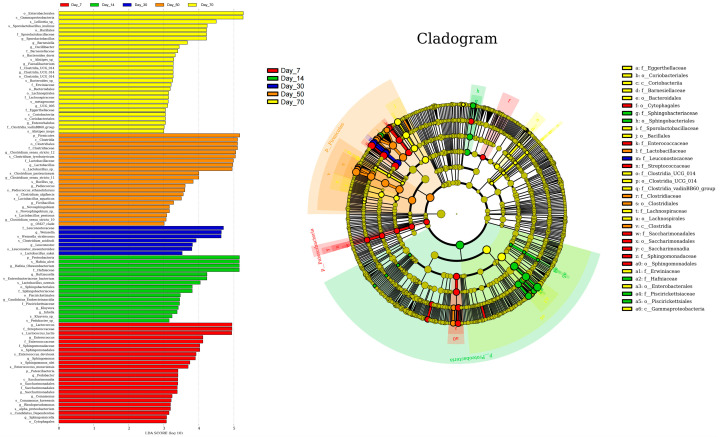
Bacterial community variations in winter wheat silage during fermentation at 7, 14, 30, 50, and 70 days, as identified by linear discriminant analysis (LDA) effect size (LEfSe).

The linear discriminant analysis (LDA) effect size (LEfSe) method was employed to identify the most differentially abundant bacterial genera in winter wheat silage across various fermentation time points (LDA score > 3.0; Figure 6). Bacterial diversity exhibited an initial decline followed by an increase as the ensiling period progressed, with the least variation in community composition observed at day 30 compared to other time points. At day 7, *Lactococcus* and *Enterococcus* were the most differentially abundant genera. Similarly, *Hafnia-Obesumbacterium* and *Buttiauxella* showed the highest differential abundance at day 14, while *Weissella* was the most distinct at day 30. By day 50 of ensiling, *Clostridium_sensu_stricto_1*, *Lactobacillus*, and *Clostridium_sensu_stricto_11* had become the most significantly differentially abundant taxa (LDA > 4.0, *p* < 0.01). This microbial profile further evolved by day 70, with *Sporolactobacillus* emerging as the most distinct biomarker (LDA = 4.5, *p* < 0.001) for winter wheat silage at the terminal fermentation stage, demonstrating its potential as a reliable indicator for monitoring silage maturation.

**Figure 7:**
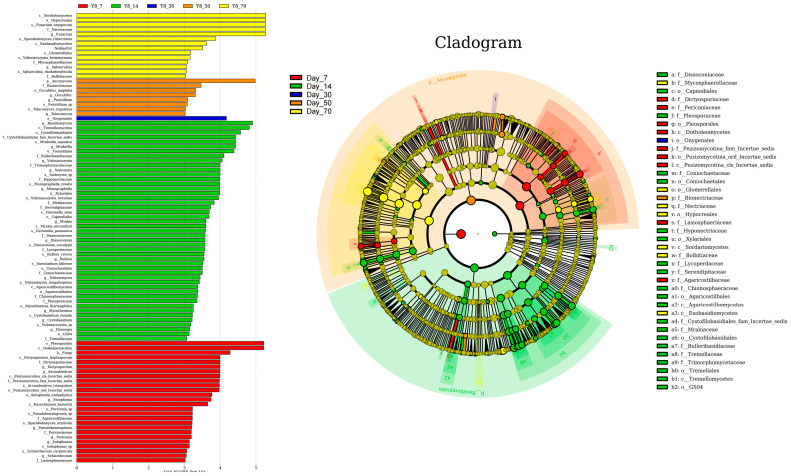
Fungal community variations in winter wheat silage during fermentation at 7, 14, 30, 50, and 70 days, as identified by linear discriminant analysis (LDA) effect size (LEfSe).

The linear discriminant analysis (LDA) effect size (LEfSe) method was employed to identify the most differentially abundant fungal genera in winter wheat silage across various fermentation time points (LDA score > 3.0; Figure 7). Similar to the bacterial community, the fungal community exhibited the highest stability at day 30. At day 7, *Dictyosporium* and *Arcuadendron* were the most differentially abundant genera, with LDA scores exceeding 4.0. By day 14, *Mrakiella*, *Vishniacozyma*, *Saitozyma*, and *Monographella* showed the highest differential abundance, while *Fusarium* emerged as the most differentially abundant genus at day 70, all with LDA scores surpassing 4.0.

### 3.4. Correlation Analysis Between Fermentation Quality and Microbial Community

**Figure 8:**
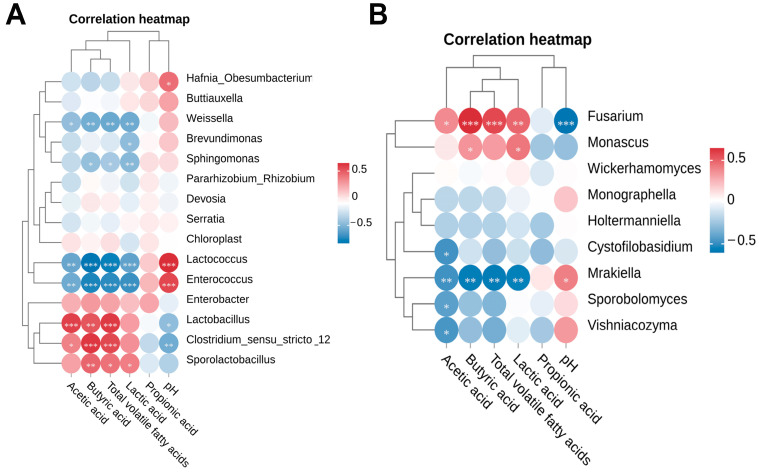
Spearman correlation analysis between fermentation quality and microbial community composition. Only bacterial (**A**) and fungal (**B**) taxa with a relative abundance exceeding 1% were included in the analysis. Significance levels are denoted as follows: * *p* < 0.05, ** *p* < 0.01, and *** *p* < 0.001.

The correlation analysis between fermentation quality parameters and the relative abundance of bacterial or fungal communities in winter wheat silage is presented in Figure 8. pH was negatively correlated with lactic acid (*R* = −0.54, *p* = 0.001), acetic acid (*R* = −0.50, *p* = 0.005), and butyric acid (*R* = −0.69, *p* < 0.001) concentrations, but positively correlated with *Hafnia_Obesumbacterium* (*R* = 0.45, *p* = 0.013), *Lactococcus* (*R* = 0.66, *p* < 0.001), *Enterococcus* (*R* = 0.58, *p* < 0.001), and *Mrakiella* (*R* = 0.43, *p* = 0.018). Conversely, pH was negatively correlated with *Lactobacillus* (*R* = −0.44, *p* = 0.015), *Clostridium_sensu_stricto_12* (*R* = −0.54, *p* = 0.002), and *Fusarium* (*R* = −0.59, *p* < 0.001). Lactic acid concentration was positively correlated with acetic acid (*R* = 0.50, *p* = 0.005), butyric acid (*R* = 0.66, *p* < 0.001), and *Fusarium* (*R* = 0.50, *p* = 0.004), but negatively correlated with *Enterococcus* (*R* = −0.68, *p* < 0.001) and *Brevundimonas* (*R* = −0.44, *p* = 0.013). Acetic acid concentration was positively correlated with butyric acid (*R* = 0.53, *p* = 0.005), total VFAs (*R* = 0.86, *p* < 0.001), *Lactobacillus* (*R* = 0.60, *p* < 0.001), *Clostridium_sensu_stricto_12* (*R* = 0.38, *p* = 0.038), and *Fusarium* (*R* = 0.39, *p* = 0.031), but negatively correlated with *Lactococcus* (*R* = −0.57, *p* = 0.001), *Weissella* (*R* = −0.44, *p* = 0.015), *Enterococcus* (*R* = −0.54, *p* = 0.001), *Mrakiella* (*R* = −0.47, *p* = 0.009), *Sporobolomyces* (*R* = −0.41, *p* = 0.024), *Vishniacozyma* (*R* = −0.44, *p* = 0.013), and *Cystofilobasidium* (*R* = −0.46, *p* = 0.010). Butyric acid concentration was positively correlated with total VFAs (*R* = 0.92, *p* < 0.001), *Lactobacillus* (*R* = 0.54, *p* = 0.004), *Clostridium_sensu_stricto_12* (*R* = 0.64, *p* < 0.001), *Sporolactobacillus* (*R* = 0.48, *p* = 0.007), *Fusarium* (*R* = 0.66, *p* < 0.001), and *Monascus* (*R* = 0.37, *p* = 0.045), but negatively correlated with *Lactococcus* (*R* = −0.81, *p* < 0.001), *Weissella* (*R* = −0.53, *p* = 0.002), *Enterococcus* (*R* = −0.74, *p* < 0.001), *Sphingomonas* (*R* = −0.42, *p* = 0.019), and *Mrakiella* (*R* = −0.56, *p* = 0.001). Total VFAs were negatively correlated with *Weissella* (*R* = −0.56, *p* = 0.001).

## 4. Discussion

Whole winter wheat exhibits remarkable potential as a high-quality feed resource, with its ensiled form providing distinct advantages in alleviating feed shortages and overcoming the challenges associated with low-quality forage for ruminants [27]. Packed with essential nutrients, including protein, carbohydrates, ether extract, and minerals, whole winter wheat silage delivers a well-rounded and balanced nutritional profile, making it an optimal feed choice for livestock [28]. Lactic acid bacteria metabolism generates organic acids during ensiling, which both protects feed nutritional quality and improves its digestibility and palatability [29,30]. Consequently, silage stands out as an ideal approach for processing and preserving winter wheat, offering a dependable and efficient means to sustain its nutritional value.

### 4.1. Changes in the Chemical Composition of Whole Wheat Silage

The quality of silage is influenced by multiple factors, including temperature, duration, raw material composition, moisture content, and the use of additives [31,32,33,34]. Optimal moisture content is critical for high-quality silage, as wilted materials exhibit poor aerobic stability, and reduced moisture levels can increase pH while diminishing the production of organic acids [35,36]. A moisture content ranging between 60% and 72% is particularly conducive to the fermentation activity of lactic acid bacteria, creating an ideal environment for ensiling [37]. In this study, the freshly harvested whole winter wheat had a moisture content of 71.31%, which likely explains why whole-plant fermented silage can be directly ensiled without additional pretreatment. Consistent with previous reports [38,39], the moisture content during natural fermentation showed minor fluctuations over time, with the dry matter content in this study reaching its maximum value by the 30th day. The initial moisture content of silage significantly influences the fermentation process, as evidenced by the slower reduction rate of NDF during the ensiling of *Stylosanthes* at 72% initial moisture compared to 60%, while the trend for ADF was the opposite [40]. The content of NDF decreased as ensiling time progressed, while ADF showed no significant change in this study, a phenomenon likely attributed to differences in initial moisture content and substrate composition. During the ensiling process, the generation of volatile substances such as carbon dioxide and methane results in a progressive decline in organic matter content [41], which aligns with the findings of this study. Altogether, whole winter wheat silage demonstrated the capacity to preserve the nutritional properties of the entire material across various storage durations, with certain attributes even exhibiting enhancement.

### 4.2. Changes in the pH and Organic Acid Content of Whole Wheat Silage

The core principle of silage technology hinges on the epiphytic microbial community, especially lactic acid bacteria, which generate organic acids that reduce pH, effectively suppressing harmful microorganism activity and safeguarding the raw materials against spoilage [42,43]. As anticipated, a gradual increase in the concentrations of lactic acid, acetic acid, and butyric acid was observed as the ensiling process progressed in this study, alongside a corresponding decline in pH. The pH value of silage, especially the benchmark of 4.2 for high-moisture silage, is critical for ensuring thorough fermentation, good aerobic stability, and long-term preservation, with lower pH values typically indicating higher quality [44]. In comparison to natural fermentation, incorporating lactic acid bacteria significantly lowered the pH value within a 60-day ensiling period [38,40]. The pH of fresh alfalfa remained above 4.5 by the 90th day of ensiling, irrespective of the addition of exogenous lactic acid bacteria [45]. However, whether broad bean leaves were used alone or mixed with wheat and oats in varying ratios, the pH value decreased to approximately 4.2 by the 60th day of ensiling [19], demonstrating that the pH during the ensiling process is heavily dependent on the raw materials. In this study, the pH of winter wheat silage was below 4.4 by the 50th day, possibly due to insufficient ensiling duration. Moreover, it is noteworthy that lactic acid and total VFAs (the main drivers of pH reduction) exhibited a continuous increase in the latter phase of the 70-day ensiling process. These findings suggest that microbial fermentation in the winter wheat silage of this study was still actively ongoing by the 70th day.

### 4.3. Microbial Diversity and Correlation

The alpha diversity serves as a measure to evaluate the richness, diversity, and evenness of species within bacterial communities [46]. In this study, the diversity and richness of fungi declined as fermentation progressed, a phenomenon likely due to the inhibitory effect of decreasing pH on fungal growth. In contrast, for bacteria, the variations in alpha diversity throughout the fermentation process were minimal in this study, with an initial reduction followed by an increase observed during the 120-day ensiling of faba beans [39]. The dominant bacterial phyla are key determinants of silage quality, and analyzing the changes in fermentation parameters and microbial composition during the ensiling process provides insights into the mechanisms of ensiling and enhances silage quality [47,48]. In this study, Proteobacteria emerged as the most abundant bacterial phylum in whole-plant winter wheat silage samples at 7, 14, 30, and 70 days. Proteobacteria play a crucial role in organic matter degradation and carbon-nitrogen cycling during anaerobic digestion [49]. However, by day 50, Firmicutes had replaced Proteobacteria as the dominant phylum, a shift likely driven by the greater adaptability of Firmicutes to acidic and anaerobic conditions [50]. The shift in the relative abundance of Firmicutes and Proteobacteria at day 70 was not an anticipated outcome, and a similar phenomenon was observed in whole-plant soybean silage over a 90-day period [51]. Ascomycota, the largest phylum in the fungal kingdom, consistently dominated the microbial community during the ensiling process of winter wheat in this study. While Ascomycota are known for their ability to decompose organic matter and degrade soil residues [52], and despite their significant presence in the microbial populations of various silage types, their precise role in the fermentation process remains poorly understood.

At the bacterial genus level, significant temporal variations in differentially abundant genera were observed throughout the experimental period. Statistical analysis identified *Lactococcus* and *Enterococcus* as the primary differential genera at day 7. However, microbial community analysis revealed a distinct shift by day 50, with *Clostridium_sensu_stricto_1*, *Lactobacillus*, and *Clostridium_sensu_stricto_11* showing significant differential abundance. *Enterococcus* demonstrates remarkable environmental adaptability, capable of surviving for 48 h across a broad pH range from 4.8 to 9.6 [53]. However, this study revealed that during the silage fermentation process, as the environmental pH continued to decrease, the population dominance of *Enterococcus* gradually diminished, with its relative abundance significantly reduced. *Lactococcus* and *Lactobacillus*, two important genera within the lactic acid bacteria group, both possess the biological capability to decompose carbohydrates into lactic acid. However, *Lactobacillus* exhibits a broader metabolic capacity, not only producing lactic acid but also synthesizing various metabolites such as acetic acid and ethanol [54]. Particularly noteworthy is that *Lactobacillus* demonstrates significantly stronger adaptability to environmental stress compared to *Lactococcus*, especially in terms of its tolerance to low pH conditions [55] This physiological characteristic explains the succession mechanism by which *Lactobacillus* displaces *Lactococcus* as the dominant microbial population during the later stages of ensiling, with its abundance variation being pH-dependent. Interestingly, at day 70, *Sporolactobacillus* emerged as the most significantly differential genus. *Sporolactobacillus* belongs to the lactic acid bacteria group (significant positive correlations with the concentrations of acetic acid, butyric acid, and total VFAs), and its functional characteristics and environmental tolerance are similar to those of *Lactobacillus* [56]. As a result, it demonstrates stronger growth advantages at 70 days of ensiling compared to earlier stages. *Fusarium* synthesizes diverse mycotoxins, including alkaloids, peptides, amides, terpenes, quinones, and pyranones [57]. Although *Fusarium* remained a dominant fungal genus during the later stages of ensiling, its growth was potentially inhibited, as evidenced by the significant reduction in overall fungal diversity.

The limitation of this study lies in the absence of absolute quantitative analysis of bacterial populations both prior to and during the ensiling process, which precludes the determination of actual bacterial abundance and may affect the interpretation of relative abundance data.

## 5. Conclusions

In summary, this study systematically investigated the dynamic changes in chemical composition, pH values, organic acid content, and microbial community structure during the 70-day ensiling process of whole-plant winter wheat. The results demonstrated that a 70-day ensiling period is insufficient to achieve stable silage products. Notably, *Sporolactobacillus* was identified as a characteristic indicator bacterium during the late ensiling stage, whose potential application as a silage additive warrants further investigation to optimize the ensiling process of whole-plant winter wheat.

## Figures and Tables

**Table 1:** Change in soil properties before and after winter wheat planting, mg/kg.

Items ^1^	pH	SOM	TN	TP	TK	AN	AP	AK
Before planting	8.24	21.40	1.06	0.55	2.12	0.10	0.13	0.19
After planting	8.05	26.60	1.67	0.76	1.72	0.11	0.39	0.12

^1^ Soil organic matter (SOM), total and available nitrogen (TN and AN), total and available phosphorus (TP and AP), and total and available potassium (TK and AK).

## Data Availability

All data generated or analyzed used in this study are available from the corresponding author upon request. Sequences of the study are available at the Sequence Read Archive (SRA) of the NCBI nucleotide database (Accession: PRJNA1241476).
